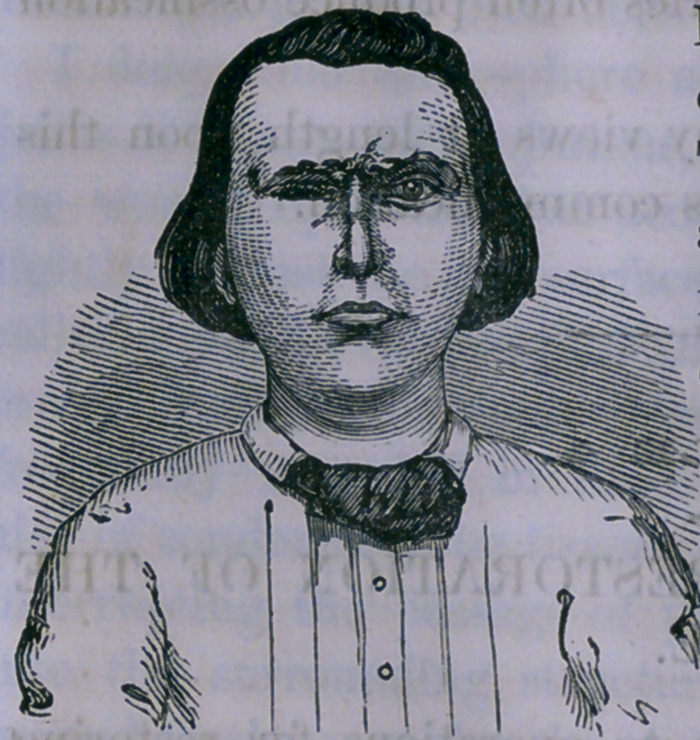# Operations for the Restoration of the Nose

**Published:** 1860-01

**Authors:** 


### ARTICLE 4.

### OPERATIONS FOR THE RESTORATION OF THE NOSE.

*Rhinoplasty*, the name given to operations for restoring the nose when lost, or improving its form when deformed, is carefully described in all recent works on operative surgery. Yet it is performed by but few surgeons, and not demanded by one patient in ten of those to whom it is applicable, from its not being sufficiently well known and favorably appreciated. Velpeau, in his Operative Surgery, speaks of it in a manner calculated to discourage its repetition, calling it "patching," and saying that it is only in some cases that it offers all the regularity desirable. Under the belief that it is extremely liable to failure, and not very satisfactory when successful, I have several times dissuaded patients from having operations of this kind performed, wherein I now believe good and satisfactory results might have been obtained. In cases of disease the chance of failure is greater than where the deformity results from injury. In these latter, with suitable skill, it will rarely fail.

The method to be chosen should be adapted to the peculiarities of each case. When the point of the organ has been lost, taking the tissue of repair from the forehead is best; when the nose has settled from ulceration of the bones and cartilages, the method recommended by Mr. Ferguson, analogous to that of Dieffenbach is sufficient.

In illustration of the former plan, the following case is given. The figure is from an ambrotype, and represents the appearance of the nose as restored with fidelity:

CASE 1. John Cullen, an Irishman, aged 23 years, had his nose bitten off and one of his eyes gouged out in an affray, on Thanksgiving day, 1856. Nearly all the cartilagious part had been removed, and the portions of the *alæ nasi* remaining were drawn asunder so as, together with the loss of the eye, to give to the countenance an expression quite hideous.

The operation was performed May 12, 1857, by bringing down a flap from the forehead in the usual manner.

The flesh was carefully adjusted to the denuded edges, and the edges of the wound occasioned by its removal brought together by stitches. The stitches employed in fixing the flesh in its place, were removed on the sixth day. Compresses and adhesive plaster being resorted to, to prevent the danger of displacement. The connection of the flap was divided June 20, the cicatrization at the points of attachment being, at that time, complete. The figure was taken three months after the operation. No one not aware of what had been done would be able to perceive at all that the nose was not natural.

CASE 2. Miss S., a very interesting young lady eighteen years of age, consulted me on account of a flatness of the nose, produced by a blow received in early childhood. The bridge of the nose was almost on a level with the face, and the lower part of it much turned to one side. One nostril was quite closed by the deviation of the septum.

The operation performed consisted in first dividing the septum and then carrying a narrow bistoury between the skin and the bones of the face from within the nose, so as to separate the tissue on the dorsum and sides of the nose, and from the cheek as far as the *infra orbitary foramen* on each side. The skin thus loosened was then transfixed from the inside with two needles armed with double ligatures, which were tied on each side over a piece of wood, the two pieces being thus approximated so as to raise the skin to the desired point. This operation succeeded perfectly, and the result was all that could be desired.

CASE 3. Recently we have performed this operation on a young man whose nose had been shockingly mangled by the bite of a dog, and which, although treated with care at the time, was so irregular after cicatrization, as to cause him to desire an operation. Although but recently performed, the result of this operation seems favorable.

There is one point, however, to which I would call attention. When the stitches require to remain many days they partially cut out, (even when silver wire is used, as was done in this instance,) and allow the skin to settle down. Cicatrices are also left, more or less unsightly. To obviate these inconveniences I removed the wires and kept the pieces of wood on each side of the nose in place by little springs of steel wire, like the *serre fine* of Vidal. This succeeded perfectly, and by proper precautions may be made to supersede the stitches altogether. Possibly they might be so employed as in case of ulcerations to prevent, in some useful degree, the deformity, and thus render any operation unnecessary.

## Figures and Tables

**Figure f1:**